# Case Report: Multiple Chromosomal Translocations Including Novel CIITA-CREBBP Fusion and Mutations in a Follicular Lymphoma

**DOI:** 10.3389/fonc.2021.620435

**Published:** 2021-03-10

**Authors:** Huan-You Wang, Ethan S. Sokol, Aaron M. Goodman, Andrew L. Feldman, Carolyn M. Mulroney

**Affiliations:** ^1^Division of Laboratory and Genomic Medicine, Department of Pathology, University of California San Diego Health System, La Jolla, CA, United States; ^2^Foundation Medicine, Cambridge, MA, United States; ^3^Division of Blood and Bone Marrow Transplant, Department of Medicine, University of California San Diego Health System, La Jolla, CA, United States; ^4^Department of Laboratory Medicine and Pathology, Mayo Clinic, Rochester, MN, United States

**Keywords:** CIITA, CIITA-CREBBP, follicular lymphoma, BCL 2, CREBBP, TBL1XR1-TP63

## Abstract

The pathogenesis of follicular lymphoma is a multi-step process, in which chromosomal translocation between immunoglobulin heavy chain (IgH) and anti-apoptotic B-cell lymphoma 2 (BCL2), namely IgH-BCL2, is an earliest step, followed by other genetic/genomic alterations including but not limited to mutation of CREB binding protein (CREBBP). MHC class II transactivator (CIITA) is a transcription regulator responsible for expression of MHC class II molecules including HLA-DR in human. We report herein a novel fusion gene involving CIITA and CREBBP in a patient with a low-grade follicular lymphoma (FL) but with high Ki-67 proliferation index. In addition, our patient also harbors CREBBP mutation. Together, we postulate that total loss of CREBBP function may contribute, in part, to the lymphoma genesis. Furthermore, this patient has addition rare (TBL1XR1-TP63) and common (IgH-BCL2) chromosomal translocations and multiple mutations including *BCL2, BRAF, MUTYH*, and *STAT6*.

## Introduction

Non-random, balanced, and less commonly unbalanced translocations involving immunoglobulin heavy chain (IgH) and less frequently light chains kappa (IgK) and lambda (IgL) with another oncogene / transcription factor are common in B-cell lymphomas, and are the contributing factor in cancer transformation as well-demonstrated in *S cerevisiae* (1, 2); however, translocations involving two oncogenes / transcription factors are rare in lymphoma in comparison to acute myeloid leukemias (AML) in either human (3) or yeast (4). Examples of balanced translocations in B-cell lymphomas include t(14; 18) involving *IgH-BCL2* in follicular lymphoma (FL) (5) and t(8; 14) involving *MYC-IgH* in Burkitt lymphoma (6). The major histocompatibility complex (MHC) class II transactivator (CIITA) is a master transcriptional regulator of diverse genes expressions beyond MHC class II and class I (7). CIITA is constitutively expressed in B-cells (8), and knock-down of CIITA or mutated CIITA in B-cells results in the absence of MHC-DR expression in the B-cell line L23 from swine (9). CREBBP, which binds to cAMP-response element-binding protein (CREB), also known as CBP short for CREB Binding Protein, is a lysine acetyltransferase functioning as one of the components of master coactivator along with p300. Although CIITA fusion with BX648577 in classical Hodgkin lymphoma (cHL) and primary mediastinal large B-cell lymphoma (PMBL) (10), and CREBBP fusions with other genes in AML (11) have been reported, CIITA fusion with CREBBP, namely CIITA-CREBBP, has not yet been reported in the English literature to the best of our knowledge. Herein we report a novel CIITA-CREBBP fusion in a histological low-grade FL but with high Ki-67 proliferation index. In addition to CIITA-CREBBP, the case reported here has several additional interesting molecular and clinical features. First, in contrast to CIITA-BX648577 fusions detected in cHL and PMBL where CIITA lost its function with resultant lack of HLA-DR expression among the lymphoma cells, to the contrary, the CIITA in CIITA-CREBBP from our case is predicted to retain its function but with predicted loss of function of CREBBP; secondly, this case harbors other two balanced translocations, namely the commonly encountered signature t(14; 18) involving IgH-BCL2 and a very rarely reported TBL1XR1-TP63; thirdly, this case exhibits multiple mutations including *BCL2, BRAF, CREBBP, MUTYH*, and *STAT6*; finally this lymphoma is a low-grade FL based on histology but shows high Ki-67 proliferation index and early clinical relapse.

## Clinical Summary

A 41-year old woman presented with extensive and bulky abdominal lymphadenopathy with small bowel involvement by large B-cell lymphoma (DLBCL) (~20–30%) arising in association with grade 3b FL (~70–80%) in early February 2018. She was treated on a clinical trial with R-CHOP [rituximab, cyclophosphamide, hydroxydaunomycin, oncovin (vincristine), and prednisone] and Pembrolizumab for six cycles, which was started right after the diagnosis and was completed by June 2018. Her first post treatment PET/CT scan was concerning for residual abdominal focus of disease, however, it was subsequently resolved without further intervention and she achieved a complete metabolic response by PET/CT in September of 2018. In November 2018 she developed back pain resembling prior symptoms of lymphoma and eventually underwent additional evaluations including two additional biopsies: an excisional biopsy from mesenteric lymph nodes in early March of 2019, which showed grade 1 FL with high Ki-67 (see below), and a core biopsy from abdomen in early June 2019, again showing grade 1 FL with high Ki-67 (not shown). A bone marrow biopsy at the same time with the early June 2019 abdominal tissue core biopsy showed no lymphomatous involvement. She was further treated with three cycles of R-ICE (rituximan, ifosfamide, carboplatin, and etoposide) from 6/29/2019 to 8/9/2019. Shortly after receiving BEAM conditioning in September 2019, she underwent autologous hematopoietic stem cell transplant, and she remains remission as of today. The patient had not received radiation therapy at all.

## Pathologic and Molecular Findings

**Figure 1:**
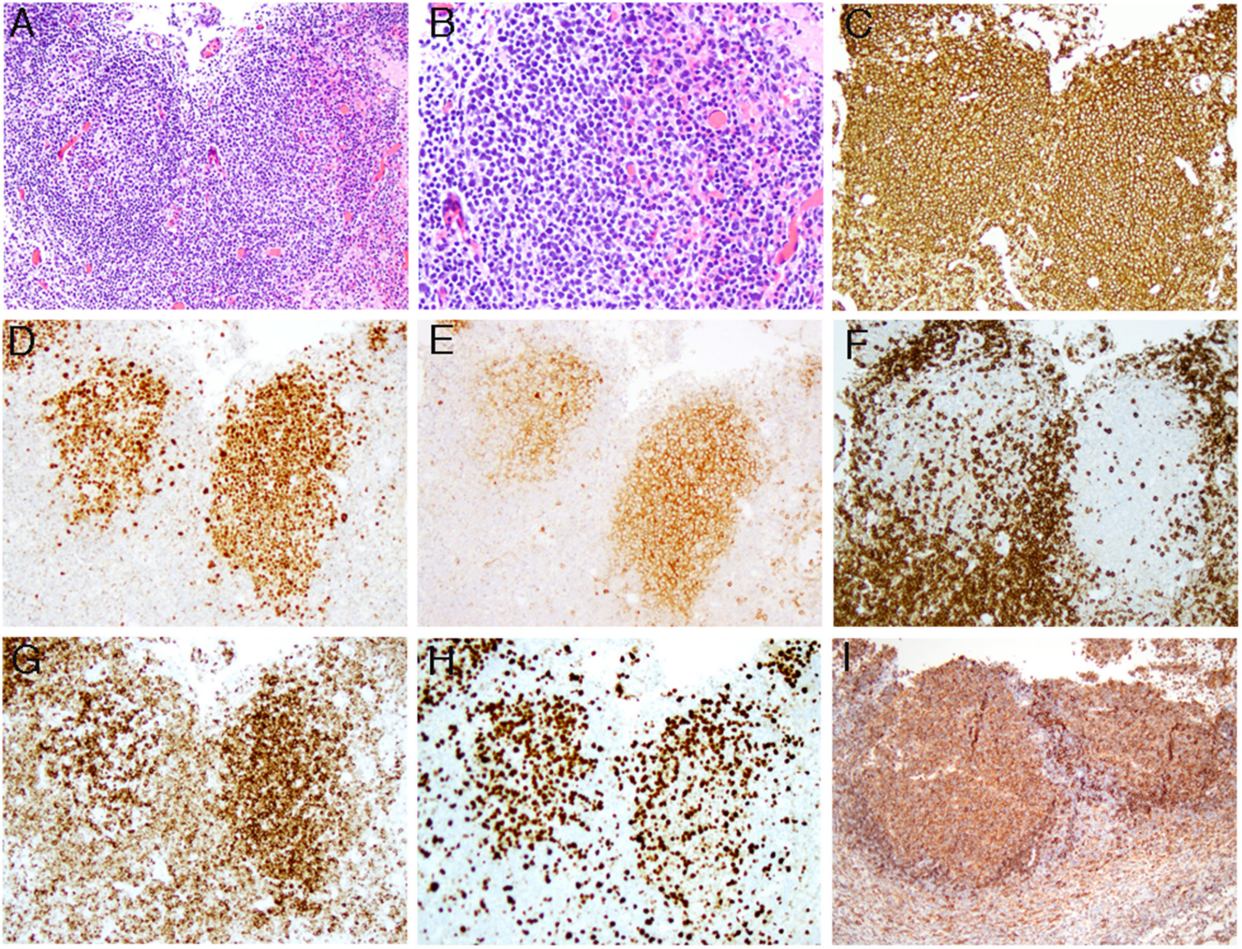
The composite microphotographs of low-grade FL with high proliferation index. **(A,B)** The follicular lymphoma cells are mainly centrocytes with <5 centroblasts per high power field [the original magnifications of **(A,B)** are 100x and 200x, respectively]; **(C–E)** the lymphoma cells are positive for CD20 **(C)**, BCL6 **(D)**, and CD10 **(E)** [the original magnifications of **(C–E)** are 100x, 100x, and 100x, respectively]; **(F–H)** In comparison to negative CD3 **(F)**, the lymphoma cells are positive for BCL2 **(G)** with high Ki-67 proliferation index **(H)** [the original magnifications for **(F–H)** are 100x, 100x, and 100x, respectively]; **(I)** The lymphoma cells are positive for HLA-DR (the original magnification is 100x).

H&E of the excised mesenteric lymph node from early March 2019 showed total effacement of architecture by similarly sized and shaped neoplastic secondary follicles with <5 centroblasts per high power field, thus consistent with a grade 1 FL (Figures 1A,B, the original magnifications of A and B are 100X and 200X, respectively). The neoplastic cells within the secondary follicles are positive for CD20 **(1C)**, CD10 **(1D)**, and BCL6 **(1E)** (the original magnifications of C-E are 100X, 100x, and 100X, respectively). In comparison to negative CD3 **(1F)** (original magnification of 100X), the neoplastic cells show co-expression of BCL2 **(1G)** (original magnification of 100X), thus confirming the morphologic impression of FL; However, Ki-67 proliferation index was high (>30%) (Figure 1H) (original magnification of 100X), therefore a final diagnosis of histologically grade 1 FL with high Ki-67 proliferation index was rendered. The neoplastic cells are also positive for HLA-DR **(1I)** (original magnification of 100X).

**Figure 2:**
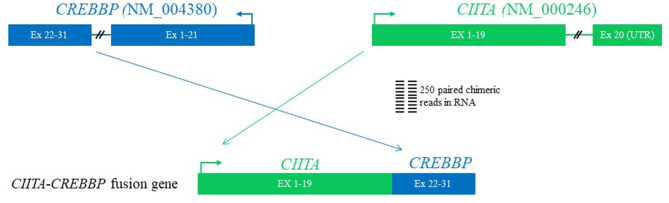
The diagram of CIITA-CREBBP fusion by next generation sequencing using formalin-fixed paraffin-embedded tissue. The first 19 exons of CIITA are fused with exons of 22–31 of CREBBP. Since exon 19 of CIITA contains a stop codon and a portion of 3′-untranslated region (UTR) at 5′ of exon 22 of CREBBP on the RNA, the fusion is postulated to generate a functional CIITA but a non-functional CREBBP.

Karyotypic analysis by G-banding yielded no metaphase cells, and fluorescence *in situ* hybridization was not performed. Comprehensive genomic profiling employing next generation sequencing (NGS) using formalin-fixed paraffin-embedded (FFPE) tissue block from early March 2019 at both the RNA and DNA levels performed at the Foundation Medicine (Cambridge, MA, USA), which interrogated a panel of 671 genes, showed the following chromosomal translocations: CIITA-CREBBP (Figure 2), IGH-BCL2 (not shown), and TBL1XR1-TP63 (not shown), and genes mutations (not shown): *BCL2* A60V, *BRAF* D594G, *CREBBP* R1446H, *MUTYH* Q400^*^, and *STAT6* D419G. Her tumor mutational burden was 9 Muts/Mb, designated as intermediate.

## Discussion

Comprehensive genomic profiling using NGS revealed both expected and novel chromosomal and genomic abnormalities as aforementioned above. While presence of IgH-BCL2 fusion, *CREBBP* and *STAT6* mutations are commonly seen in FL, CIITA-CREBBP has not yet been described in FL or in any lymphoma from the English literature so far, thus this is a novel finding. CREBBP and CIITA are both located at the same primary band on the short arm of chromosome 16, namely 16p13.3 and 16p13.13, respectively, thus the novel CIITA-CREBBP translocation discovered here was an intra-chromosomal translocation *via* homologous recombination through the following two mechanisms: joining of CIITA and CREBBP with resultant deletion of the secondary band 3 through 13 within the portion of primary band 13; alternatively, the homologous recombination of CIITA and CREBBP was according to the Robersonian model. Given the fact that cells carrying this translocation were lymphoma cells, the chromosome involved was 16, but not the commonly five acrocentric chromosome pairs, the first possibility that resulted in an unbalanced gene dosage are favored. The breakpoints of CIITA-CREBBP in the current case are distinct from the previously documented CIITA-BX648577 (10) in that the first 19 exons of CIITA are fused with exons 22–31 of CREBBP based on RNA read support (Figure 2). Although we do not have direct evidence to prove CIITA is functional, based on the genomic structure [exon 19 is the final coding exon of CIITA including the stop codon and a portion of the 3' untranslated region (UTR)], and presence of HLA-DR protein expression as revealed by immunohistochemistry [a surrogate marker for CIITA) among the lymphoma cells (Figure 1I)], we conclude that the CIITA-CREBBP does not affect CIITA's function. However, CREBBP in the CIITA-CREBBP fusion most likely lost its expression and function (since the RNA transcript has a stop codon and portion of the 3'-UTR at the 5' of CREBBP exon 22), thus the lymphoma cells may have no CREBBP expression at all since we also observed a somatic mutation of *CREBBP* R1446H as well. Since BCL2-IGH occurs very early in the development of FL during the V(D)J recombination (12), complete loss of function of CREBBP due to combined CIITA-CREBBP fusion and mutation is most likely an early but secondary event as well-demonstrated by Horton et al. (13). CREBBP mutations occur in ~64% of FL, and were proposed to serve as early driver mutations (13, 14). Although it is a well-known phenomenon that radiation alone or in combination with chemotherapy increases the likelihood of secondary translocations and/or mutations (15), based on the early studies in particular *CREBBP* mutations were frequently detected in *in situ* follicular neoplasm (13, 14), the *CREBBP* mutation and translocation (CIITA-CREBBP) were most likely *de novo* rather than therapy-induced secondary events because of the additional facts that the patient had never received radiation therapy, and this biopsy were performed only after the first cycle of R-CHOP chemotherapy, too soon interval to evoke such genetic event.

The presence of CIITA-CREBBP in this particular FL is worthy notice not only because of its novelty, but also because this FL, while morphologically low-grade, exhibits higher (>30%) proliferation index as demonstrated by Ki-67, thus this FL belongs to the so-called “low histologic grade FL with high proliferation index,” which has been reported to be associated with clinical behavior more akin to grade 3 FL according to the study by Wang et al. (16). More studies on the incidence of CIITA-CREBBP fusion are needed in order to assess the true relationship between the CIITA-CREBBP and low histologic grade FL with high proliferation index.

The other chromosomal translocation detected in this case, namely TBL1XR1-TP63, is reported in 5% (6/115) and 1.2% (1/81) of DLBCL and FL, respectively (17), thus this is the only second documented FL with TBL1XR1-TP63. The breakpoints of TBL1XP1 (exons 1-7) and TP63 (exons 4-14) we observe here are similar to those reported from at least one case of DLBCLs but different from the reported FL by Scott et al. (17). Of interest, TBL1XP1-TP63 is also observed rarely in peripheral T-cell lymphoma [1.1% (2/190)], where TP63 rearrangements rendered significant inferior overall survival (18).

Besides the aforementioned three fusions, this patient also harbors multiple additional mutations of several genes including *BCL2* and *STAT6*. *BCL2* mutations occur in more than 50% of the FL. BCL2 mutations including A60V as seen in this patient result in overexpression of BCL2 and undoubtedly contribute to the lymphoma growth. Unlike *BCL2* G101V, which confers resistance to *BCL2* inhibitor in chronic lymphocytic leukemia (19), *BCL2* A60V appears to have no adverse effect in overall survival and progression-free-survival on FL treated with rituximab (20). *STAT6* mutations are found in 11% of FL and are implicated as a driver of FL pathogenesis (21).

## Data Availability Statement

The original contributions presented in the study are included in the article/supplementary material, further inquiries can be directed to the corresponding author/s.

## Ethics Statement

Ethical review and approval was not required for the study on human participants in accordance with the local legislation and institutional requirements. Written informed consent for participation was not required for this study in accordance with the national legislation and the institutional requirements.

## Author Contributions

HYW wrote the draft, took microphotographs, made Figure 1, and finalized the revision. ESS performed NGS and made Figure 2. AMG and CMM took care of the patient. ALF performed HLA-DR immunohistochemistry. All authors contributed to the article and approved the submitted version.

## Conflict of Interest

AG is a consultant for Seattle Genetics and EUSA Pharma. The remaining authors declare that the research was conducted in the absence of any commercial or financial relationships that could be construed as a potential conflict of interest.
